# Photoelectrochemical biosensor based on SiW_12_@CdS quantum dots for the highly sensitive detection of HPV 16 DNA

**DOI:** 10.3389/fbioe.2023.1193052

**Published:** 2023-06-14

**Authors:** Yao Cheng, Chaoyue Sun, Yuhua Chang, Jiayin Wu, Zhihao Zhang, Yunqing Liu, Shenguang Ge, Zhao Li, Xiao Li, Liang Sun, Dejin Zang

**Affiliations:** ^1^ National Key Laboratory of Advanced Drug Delivery and Release System, NHC Key Laboratory of Biotechnology Drugs (Shandong Academy of Medical Sciences), Key Lab for Rare and Uncommon Diseases of Shandong Province, School of Pharmacy and Pharmaceutical Sciences, Institute of Materia Medica, Shandong First Medical University and Shandong Academy of Medical Sciences, Jinan, China; ^2^ Institute for Advanced Interdisciplinary Research (iAIR), School of Chemistry and Chemical Engineering, University of Jinan, Jinan, China; ^3^ Shandong Provincial Maternal and Child Healthcare Hospital, Jinan, China; ^4^ Suzhou KunTao Intelligent Manufacturing Technology Co., Ltd., Suzhou, China; ^5^ NMPA Key Laboratory for Quality Evaluation of Medical Materials and Biological Protective Devices, Jinan, China; ^6^ Shandong Institute of Medical Device and Pharmaceutical Packaging Inspection, Jinan, China

**Keywords:** biosensor, polyoxometalates, quantum dots, HPV 16 DNA, photoelectrochemistry

## Abstract

A highly sensitive biosensor for detecting HPV 16 DNA was prepared based on Keggin-type polyoxometalate (SiW_12_)-grafted CdS quantum dots (SiW_12_@CdS QDs) and colloidal gold nanoparticles (Au NPs), which exhibited remarkable selectivity and sensitivity upon target DNA detection because of its excellent photoelectrochemical (PEC) response. Here, an enhanced photoelectronic response ability was achieved with the strong association of SiW_12_@CdS QDs by polyoxometalate modification, which was developed through a convenient hydrothermal process. Furthermore, on Au NP-modified indium tin oxide slides, a multiple-site tripodal DNA walker sensing platform coupled with T7 exonuclease was successfully fabricated with SiW_12_@CdS QDs/NP DNA as a probe for detecting HPV 16 DNA. Due to the remarkable conductivity of Au NPs, the photosensitivity of the as-prepared biosensor was improved in an $I_{3}^{-}/I^{-}$ solution and avoided the use of other regents toxic to living organisms. Finally, under optimized conditions, the as-prepared biosensor protocol demonstrated wide linear ranges (15–130 nM), with a limit of detection of 0.8 nM and high selectivity, stability, and reproducibility. Moreover, the proposed PEC biosensor platform offers a reliable pathway for detecting other biological molecules with nano-functional materials.

## 1 Introduction

Cancer has always been the most malignant disease affecting human health, with high morbidity and mortality rates. The development of targeted diagnosis and personalized treatment has never stopped; thus, early diagnosis with precise cancer biomarker recognition that will offer valuable opportunities for more effective treatment is of great significance to specific therapy of cancer patients (Garland, 1953; Helmink et al., 2019). Nowadays, various therapeutic modalities based on chemotherapy regimens have been exploited for mid–late stage cancer patients despite a lack of research into earlier diagnosis and more effective treatments. Thus, the exploration of highly effective diagnoses with remarkable sensitivity, high selectivity, and reliability remains challenging and is urgently required (Xiao et al., 2022).

Among various cancers, cervical cancer is the second most common cancer in women; moreover, cervix cancer caused by infection with high-risk human papillomavirus (HPV) accounts for more than 99% of cervical cancers. Although stage-specific survival has been improved since the 1960s, along with the development of multi-modality treatment, the 5-year survival rate of women with advanced non-metastatic cervical carcinomas is still low at ∼40%. However, the cure rate could reach 70%–85%, which would occur in cervical cancer patients with stage I and II_a_ lesions, indicating the very significance of early diagnosis (Kay et al., 2005). Two high-risk sexually transmittable human HPV types of 16 and 18 can cause cervical cancers. Importantly, this cancer shows no symptoms until the advanced stages of the disease. Therefore, finding a new diagnostic methodology that can detect the presence of HPV or cervical cancer at the earliest stage is a real challenge, which also stimulates the development of new biosensors for cancer early diagnosis (Jampasa et al., 2018).

Photoelectrochemical (PEC) biosensors based on photocurrent conversion functional materials are an ideal pathway to detect biomolecules owing to their low background signal and excellent sensitivity (Kay et al., 2005; Jampasa et al., 2018; Wang F. et al., 2022; Wang L. et al., 2022; Huang et al., 2022; Nanocubes et al., 2023). However, the unfavorable biocompatibility, belated photocurrent response, and low stability of these functional materials have limited the development of PEC biosensors. Compared with the electrochemiluminescence immunoassay strategy, which depends on the concentration of ^•^OH induced by H_2_O_2_ conversion, PEC biosensors require no auxiliary additives and exhibit lower toxicity and higher sensitivity but rely heavily on outstanding photochromic properties (Nie et al., 2020; Wang L. et al., 2022). Quantum dots (QDs) are extensively used in the fields of energy catalysis (Liu et al., 2014; Weiss, 2017; Kong et al., 2018; Shi et al., 2019; Zheng et al., 2020; Zhang M. et al., 2022), imaging (Nguyen et al., 2017; Park et al., 2017; Mallick et al., 2019; Min et al., 2019; Zheng et al., 2020; Xu et al., 2021; Liu et al., 2017), and chemical sensors (Wang F. et al., 2022; Zhang J. et al., 2022; Huang et al., 2022) due to their remarkable photoelectric response properties. Cadmium sulfide quantum dots (CdS QDs) have attracted broad and interdisciplinary attention for a long time because of their excellent properties in that their band gap (2.3 eV) corresponds well with the spectrum of sunlight, qualifying their superior visible light photosensitiveness and proposing remarkable compatibility with other functional materials (Ahamad et al., 2016). More importantly, the photoelectric properties of CdS QDs can be significantly tuned by introducing heteroatoms or dopants into their lattice or matrix. Modified CdS QDs are regarded as promising photocurrent conversion materials and have been widely used in solar cells and biological sensors (Jeong et al., 2017; Smith et al., 2017; Lee et al., 2018; Morgan and Kelley, 2018; Sui et al., 2018; Zhang et al., 2019). Multiple synthetic strategies, such as growth doping, nucleation doping, diffusion doping, and single-source precursor strategy have been reported for the synthesis of modified CdS QDs (Sui et al., 2018; Yu et al., 2021). In parallel, polyoxometalates (POMs) have emerged as a new class of materials due to their unique electronic, optical, magnetic, and catalytic properties (Luo et al., 2013; Ueda, 2018; Kong et al., 2020; Liu et al., 2020; Misra et al., 2020; Gu et al., 2021; Fabre et al., 2022; Kruse et al., 2022). According to some recent reports, when CdS QDs and POMs are successfully composited to have a hierarchical nanostructure under certain conditions, a unique phenomenon of interaction involving electron and energy transfer will occur (Xing et al., 2013; Dong et al., 2021). Such as-prepared POM@CdS QD composites demonstrate a novel strategy toward advanced photoelectric functional materials.

POMs are a class of negatively charged molecular metal oxides with well-defined structures, beautiful geometries, and nanoscale sizes (Luo et al., 2013; Xing et al., 2013; Kong et al., 2020; Kondinski, 2021) and have been successfully used in a wide domain of industrial catalysis of functional materials (Ji et al., 2015; He et al., 2016; Tourneur et al., 2019; Zang et al., 2019; Wang et al., 2020; Gul et al., 2022; Shi et al., 2022), environmental science (Girardi et al., 2015; Chen et al., 2018; Guo et al., 2018; Cao et al., 2019; Huang et al., 2019; Li C. et al., 2020; Yu H. et al., 2020; Li N. et al., 2020; Yu F. Y. et al., 2020; Lang et al., 2020; Zang et al., 2021; Fabre et al., 2022; Zang and Wang, 2022), life science (Bijelic et al., 2019; Li N. et al., 2020; Shi et al., 2020; Alizadeh and Yadollahi, 2022; Su Y. et al., 2022; Fabre et al., 2022; Xiao et al., 2022), pharmacology (Sarver et al., 2021; Su Y. et al., 2022; Liu et al., 2022), and other disciplines (Boulmier et al., 2018; Mitchell et al., 2022). As additives in the modification of CdS QDs, POMs with rich charges and excellent electron transfer ability can rationally adjust their band gaps through the synergistic effect to eliminate the intrinsic limits of their rapid recombination of photogenerated carriers and severe photocorrosion, improving the PEC performance of POM@CdS QD composites (Dong et al., 2021). Meanwhile, POM@CdS QDs are rarely applied in PEC biosensor fabrication for biomolecule detection such as protein, DNA, or RNA.

In this study, a highly sensitive biosensor for detecting HPV 16 DNA fabricated with Keggin-type POM (SiW_12_)-grafted CdS QDs (SiW_12_@CdS QDs) and colloidal gold nanoparticles (Au NPs) is reported for the first time. These SiW_12_@CdS QDs exhibited enhanced photocurrent response and high stability after being combined with NP DNA; with chitosan (CS)/Au NPs as the first layer on indium tin oxide (ITO) slides, a series of biochemical DNA primers were incubated to fabricate a multi-site tripodal DNA walker sensing platform coupled with T7 exonuclease. Due to the remarkable conductivity of Au NPs, the photosensitivity of the as-prepared biosensor was further improved in an $I_{3}^{-}/I^{-}$ solution and avoided the use of other regents toxic to living organisms. Finally, under optimized conditions, the as-prepared biosensor protocol demonstrated wide linear ranges (15–130 nM), with a limit of detection (LOD) of 0.8 nmol/L and high selectivity, stability, and reproducibility. Furthermore, the proposed PEC biosensor platform offers a reliable and promising pathway for detecting biological molecules.

## 2 Experiments

### 2.1 Materials and methods

All chemical reagents in this experiment were of analytical grade. Na_2_S·9H_2_O, CdCl_2_·2.5H_2_O, I_2_, KI, and HAuCl_4_·4H_2_O were purchased from Sinopharm Chemical Reagent Co., Ltd. (Shanghai, China). NaOH and trisodium citrate dihydrate (Na_3_C_6_H_5_O_7_·2H_2_O) were obtained from Shanghai Aladdin biochemical technology Co., Ltd. (Shanghai, China). H_4_[Si(W_3_O_10_)_4_]·xH_2_O, CS was obtained from Shanghai Maclin Biochemical Technology Co., Ltd. Synthetic oligodeoxy-nucleotides corresponding to partial sequences of the gene of HPV type 16 and TE buffer were received from Sangon Biotech (Shanghai) Co., Ltd. T7 Exo and NE buffer were received from New England Biotechnology (Beijing) Co., Ltd. HPV samples of vaginal swab scraping with different infection subtypes (HPV 16, 18, 33 DNA) were supplied by Suzhou KunTao Intelligent Manufacturing Technology Co., Ltd. All reagent solutions were prepared using ultrapure water (resistivity as 18 MΩ⋅cm at 25°C). The nucleotide sequences of the oligonucleotides are listed in Table 1.

**TABLE 1:** The nucleotide sequences of oligonucleotides.

Name	Sequence (5′ to 3′)
ArmDNA	TTTTTGCTGGAGGTTTTTTTTTTTTTTTTTTTTTTTTTTTTTTTTTTTTTTTTTTT-(CH_2_)_3_-SH
cDNA	CATACACCTCCAGC
pDNA	SH--(CH_2_)_6_-GCCGGACTAG
NP DNA	COOH-TCCAGCGGGCTAGTC
HPV 16 DNA	GCTGGAGGTGTATG
HPV 18 DNA	GGATGCTGCACCGG
HPV 33 DNA	CACATCCACCCGCA

### 2.2 Synthesis of SiW_12_@CdS QDs

First, CdS QDs were synthesized according to our previously published literature (Wang F. et al., 2022). Afterward, a post-modification procedure of CdS QDs with SiW_12_ proceeded. H_4_[Si(W_3_O_10_)_4_]·xH_2_O (0.1435 g) were dissolved in a CdS QD solution (15 mL), and the obtained mixed solution was vigorously stirred at 40°C for 12 h to obtain the final products, a homogeneous yellow solution. The product solution was stored at 4°C for the next step.

### 2.3 Preparation of Au particle-based CS hydrogel

Au NPs were synthesized according to the previously published literature (Wang F. et al., 2022). First, 1-mg CS and a 20-mL gold solution were added to 30-mL ultrapure water. After that, the obtained solution was stirred at room temperature (25°C) for 24 h to achieve a CS hydrogel. The obtained Au NP-based CS hydrogel was stored at 4°C (Suginta et al., 2013; Liu et al., 2018; Feyziazar et al., 2020; Vesel, 2023).

### 2.4 Construction of PEC biosensor

5 mL of a 9-μM armDNA solution and 5 mL of a 9-μM cDNA solution were mixed and heated at 95°C for 5 min. After cooling down to room temperature, arm-cDNA was received. For the fabrication of the PEC biosensor, 40 μL of the Au NP-based CS hydrogel (Au NP/CS gel) was embellished on the ITO slide surface, and then, 2 μL of the arm-cDNA solution and 3 μL of 3-μM pDNA were sequentially modified on the electrode. When they were successfully connected to the electrode, 3 μL of the SiW_12_@CdS QD/NP DNA solution was successively modified and incubated for 2 h, and unstable residues on the electrode surface were washed with buffer. Finally, the prepared electrode was incubated with a series of concentrations of HPV 16 DNA for 2 h. Then, the electrode was dropped in 50-U mL^−1^ T7 exonuclease and incubated for 2 h. The electrode was rinsed with buffer and dried under nitrogen atmosphere for PEC measurements in a 5-mM $I_{3}^{-}/I^{-}$ working solution. Here, an HPV 16 DNA-detecting biosensor was successfully fabricated.

### 2.5 PEC measurement procedure

The detection performance and reliability of the as-prepared PEC biosensor were investigated with the samples from vaginal swab scraping. Different specimens of the as-prepared biosensor were incubated with different patients’ samples of HPV 16 DNA for 2 h. Then, the electrode was washed and dropped in 50-U mL^−1^ T7 exonuclease. Finally, the electrode was rinsed with buffer and dried under nitrogen atmosphere for PEC measurements in 5-mmol L^−1^ of $I_{3}^{-}$ and 0.5-mol L^−1^ of an I^−^ working solution, and each sample was detected three times. Similarly, to detect the samples of HPV 18 DNA and HPV 33 DNA to evaluate the selectivity and stability of the as-prepared biosensor, the same PEC measurement procedure was performed with different HPV DNA subtypes.

### 2.6 Material characterization

Transmission electron microscopy (TEM) images of the NPs were obtained using an HT7800 transmission electron microscope at 200-kV acceleration voltages. The Zeta potentials and particle size distribution of the SiW_12_@CdS QDs were obtained by Malvern nano-ZS NP size and Zeta potential analyses, and UV–vis absorption spectra were recorded with a UV-26001 UV–vis spectrophotometer. Electrochemical data were obtained with a three-electrode-system CHI 660E electrochemical workstation. PEC signals were obtained from a PL-X500D Simulated solar xenon lamp source and electrochemical word station (CHI 660e). A single-sided glass electrode (1 cm × 3 cm) was coated with ITO as the working electrode. A platinum wire and a saturated calomel electrode were used as the counter and reference electrodes, respectively; PEC measurements were performed in a 5-mM $I_{3}^{-}/I^{-}$ working solution.

## 3 Results and discussion

**SCHEME 1:**
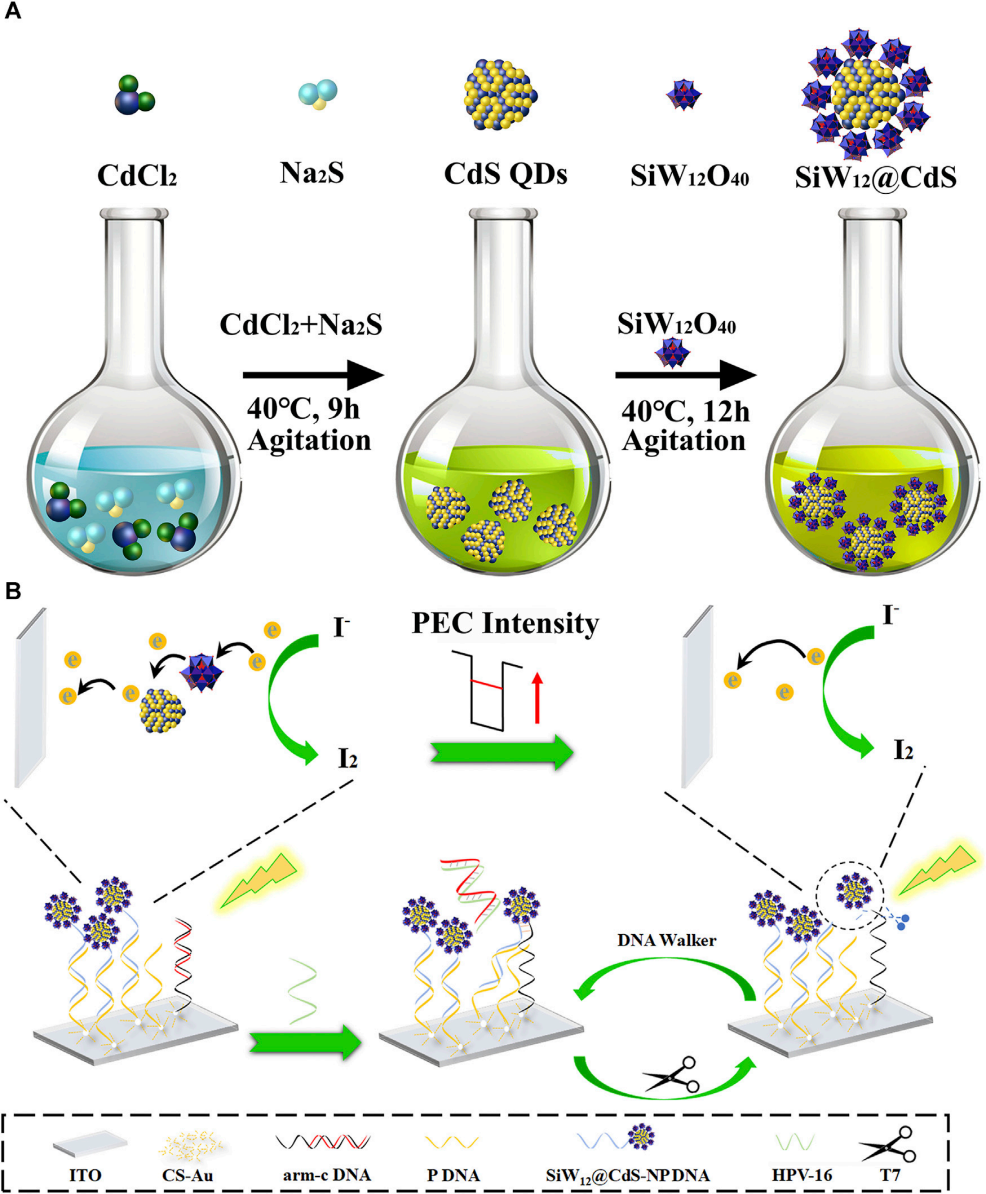
Schematic illustration of **(A)** the synthesis of POM@CdS QD composites and **(B)** PEC sensor for detecting HPV 16 DNA.

The synthesis of key photochromic materials such as SiW_12_@CdS NPs, the fabrication of the as-prepared PEC biosensor, and the comprehensive detection process of HPV DNA were performed according to the procedure shown in Scheme 1.

### 3.1 Characterization of SiW_12_@CdS QDs

**FIGURE 1:**
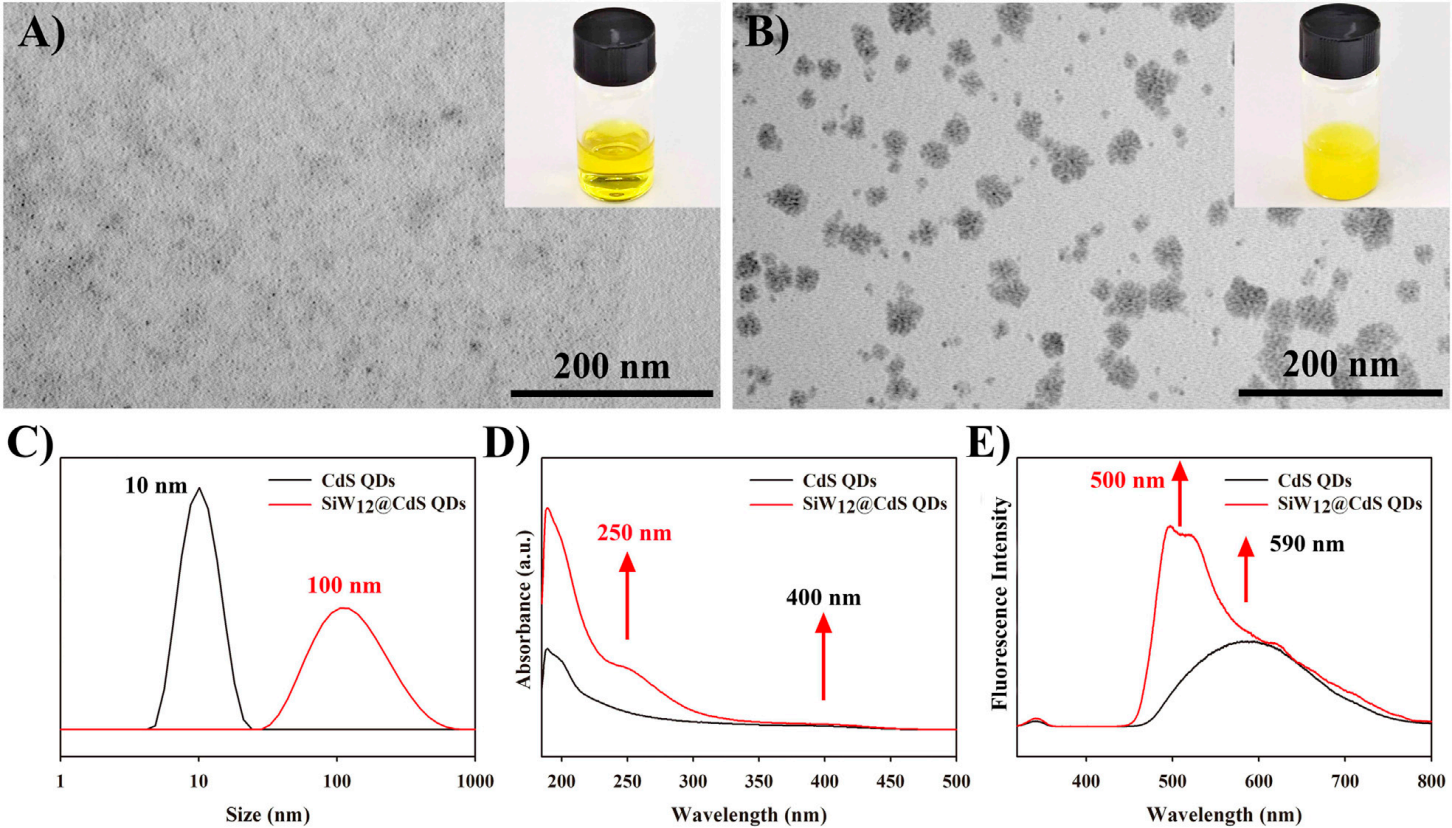
TEM images of **(A)** CdS QDs and **(B)** SiW_12_@CdS QDs. **(C)** Size distribution diagram of CdS QDs and SiW_12_@CdS QDs. **(D)** UV–vis absorption spectra of CdS QDs and SiW_12_@CdS QDs. **(E)** Fluorescence emission spectra of CdS QDs and SiW_12_@CdS QDs, excitation wavelength: 340 nm.

The morphology and size distribution of the as-prepared CdS QDs and SiW_12_@CdS NPs were characterized via TEM and Malvern nano-ZS NP size analysis. As shown in Figures 1A, C black curve, uniform CdS QDs were obtained using the solvothermal method and observed as a yellow solution (insert photograph on Figure 1A), and the average particle size of the obtained CdS QDs was ∼10 nm; note that the size of the as-prepared SiW_12_@CdS NPs has increased to ∼100 nm, making the color brighter yellow, as shown in Figures 1B, C red curve. The larger size resulted from the aggregation of the SiW_12_-modified CdS QDs, as shown in Figure 1B (Liu et al., 2018; Feyziazar et al., 2020; Zhang J. et al., 2022; Vesel, 2023). Here, the SiW_12_@CdS NPs were successfully synthesized. The photographs of the as-prepared CdS QDs and SiW_12_@CdS NPs under UV light irradiation are shown in Supplementary Figure S1.

After the modification of SiW_12_ to CdS QDs, a new absorption wave appeared at ∼250 nm on the UV–vis absorption spectrum of the SiW_12_@CdS NPs (Figure 1D, red curve) compared with that of the CdS QDs (Figure 1D, black curve), and both underwent absorption at ∼400 nm. Thus, improved light absorption was achieved according to this phenomenon. Otherwise, from Figure 1E of the fluorescence emission spectrograms of the CdS QDs and SiW_12_@CdS QDs, an enhanced emission spectrum and a 90-nm blue shift were observed from ∼590 nm of the CdS QDs to ∼500 nm of SiW_12_@CdS QDs under the same excitation light at 340 nm, indicating a strong interaction between SiW_12_ and CdS QDs with a broader band gap (Guo et al., 2016; Ji et al., 2017; Zang et al., 2022; Nie et al., 2020). To summarize the UV–vis absorption and fluorescence emission spectroscopic studies, the improved light response ability was successfully achieved by the strong association between SiW_12_ and CdS QDs, indicating remarkable photoelectric properties.

### 3.2 Characterization of Au NPs

**FIGURE 2:**
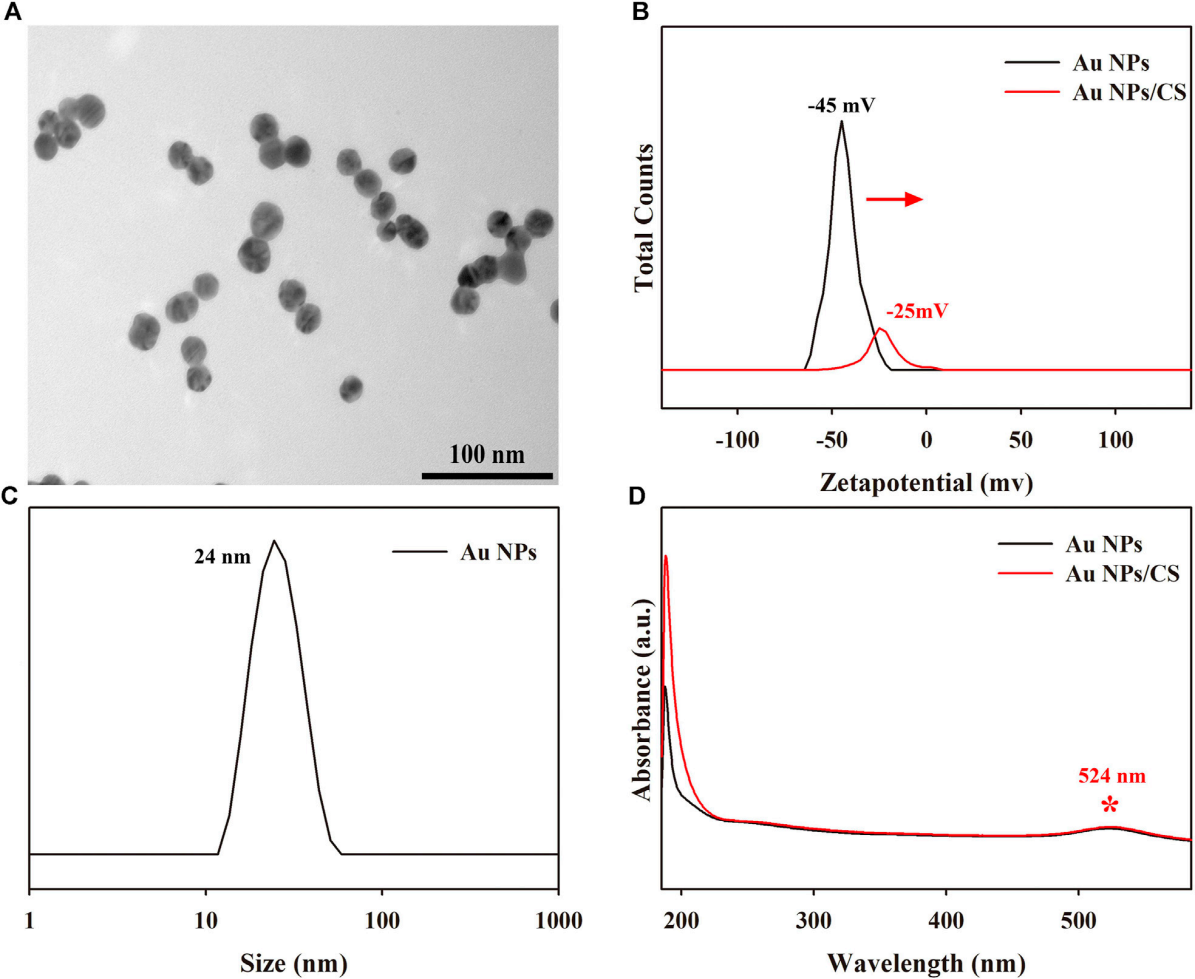
**(A)** TEM images of Au NPs. **(B)** Zeta potential distribution curves of Au NPs and Au NP/CS gel. **(C)** Size distribution diagram of Au NPs. **(D)** UV–vis absorption spectra of Au NPs and Au NP/CS gel.

As an important role of the first layer in the construction of the as-prepared PEC biosensor, Au NPs were synthesized using the solvothermal method, as shown in Figure 2A, with a size of ∼24 nm in Figure 2C. Notably, during the preparation of the Au NP/CS gel (Au NP/CS gel), the Zeta potential of the Au NPs decreased by 20 mV from −45 to −25 mV (Figure 2B), indicating a strong accumulating capacity, which would endow it with good adhesive ability as the first layer on the ITO slide to fabricate the PEC biosensor, while the visible light absorption ability of the Au NPs was well maintained without any changes in the UV–vis absorption spectrum at 524 nm (as shown in Figure 2D), high conductivity and visible light absorption for the PEC biosensor.

### 3.3 PEC characterization of PEC biosensor

**FIGURE 3:**
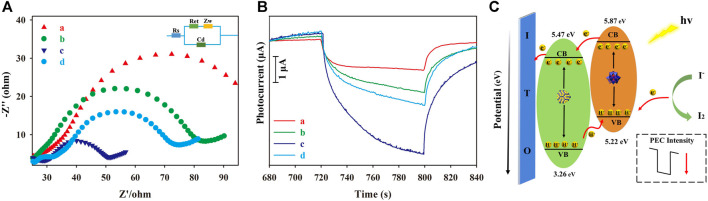
**(A)** EIS spectra of each modification of the ITO slide; **(B)** Photocurrent response of each modification of the PEC biosensor; **(C)** Schematic of the PEC mechanism of the as-prepared biosensor. (a) ITO/Au NP–CS; (b) ITO/Au NP–CS/arm-cDNA, pDNA; (c) ITO/Au NP–CS/arm-cDNA, pDNA/NP DNA–SiW_12_@CdS QDs; (d) ITO/Au NP–CS/arm-cDNA, pDNA/NP DNA–SiW_12_@CdS QDs/15-nM HPV 16. The measurements were performed under working conditions of 5 mmol L^−1^ of $I_{3}^{-}$ and 0.5 mol L^−1^ of I^−^ solution.

Electrochemical impedance spectroscopy (EIS) changes associated with the modification of the ITO slide and the PEC response of the as-prepared PEC biosensor were measured for each modification layer. As shown in Figure 3A, the first layer of the Au NP/CS gel on the ITO slide showed the largest R_et_ value (Figure 3A; a: red curve) because the poor conductivity of the CS gel obstructed electron transfer to the ITO electrode. Au NP addition not only increases conductivity but also PEC response via the LSPR effect, emphasizing its importance (Aiken and Finke, 1999; Lee et al., 2013; Chou et al., 2017; Wen et al., 2017; Domingues et al., 2018; Shi et al., 2019; Figueiredo et al., 2021). After the successive modification with arm-c DNA and pDNA (Figure 3A, b: green curve), the obtained R_et_ value decreased because of the association between Au NPs and the primers, offering an electron transfer pathway with reasonable steric hindrance to the ITO electrode. The smallest R_et_ value appeared after the NP DNA–SiW_12_@CdS NP modification (Figure 3A, c: dark blue curve), for the reason that the remarkable conductivity of the SiW_12_@CdS NPs significantly improved electron transfer. The R_et_ value was increased when the HPV 16 DNA was added, as shown in Figure 3A, d: light blue curve, owing to the high steric hindrance of HPV DNA. The photocurrent response (PEC) was consistent with the EIS investigation, as shown in Figure 3. The ITO/Au NP/CS sample exhibited the smallest photocurrent (Figure 3B, a: red curve), and the photocurrent increased when the primers were continuously anchored onto the modified ITO slide (Figure 3B, b: green curve). The largest photocurrent was achieved by the NP DNA–SiW_12_@CdS NP modification (Figure 3B, c: dark blue curve) because of the strong synergistic effect on the photoelectronic phenomenon that occurred within the association of SiW_12_@CdS NPs. Here, the photocurrent response of the as-prepared PEC biosensor with the outmost layer of NP DNA–SiW_12_@CdS NPs established the maximum photocurrent monitoring range.

From the spectrum analysis in Figures 1D, E and photocurrent response analysis in Figure 3B, the intense PEC response occurrence mechanism for the largest photocurrent with SiW_12_@CdS QDs can be illustrated, as shown in Figure 3C. The SiW_12_@CdS QDs were stimulated under Xe light irradiation, in detail. The highest occupied molecular orbital (HOMO) of both the SiW_12_ and CdS QDs were stimulated to generate photo-electrons (e^−^) and photo-holes (h^+^) simultaneously; $I_{3}^{-}/I^{-}$ electrolytes donating electrons via I^−^ to I^0^ occurred on the excited HOMO of SiW_12_ through the generated photo-holes (h^+^). The photo-electrons naturally transfer to the lowest unoccupied molecular orbital (LUMO) of SiW_12_ and then to the LUMO of CdS QDs due to the close contact between them, with a matched energy level (0.4 eV difference between 5.87 and 5.47 eV). Successfully, the photo-electrons finally moved to the ITO external circuit to generate photocurrent, and the generated photo-holes (h^+^) from the HOMO of CdS QDs are transferred to the HOMO of SiW_12_ to complete the PEC procedure (Liang et al., 2015; Kokal et al., 2016; Li et al., 2016; Shi et al., 2018; Su S. et al., 2022; Kar et al., 2023). The enhanced PEC response of SiW_12_@CdS QDs to bare CdS QDs is attributed to the increased photogenerated electron energy of 0.4 eV from the strong synergistic effect resulting from the association of SiW_12_@CdS NPs realizing the outstretched band gap from a HOMO of 3.26 eV to LUMO of 5.87 eV.

### 3.4 Detection of HPV 16 DNA

**FIGURE 4:**
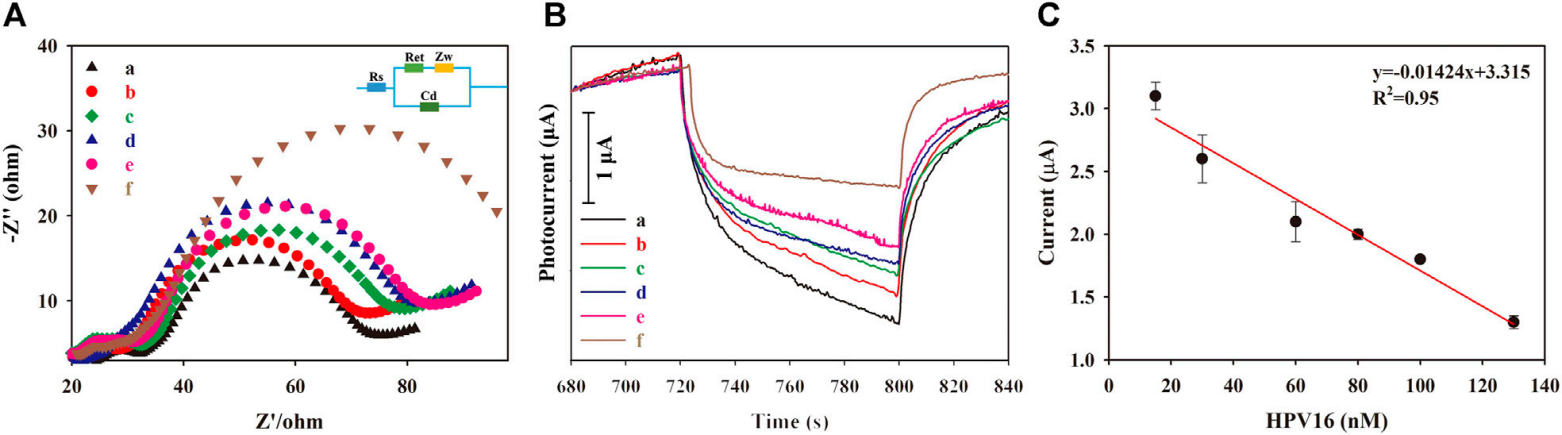
**(A)** EIS spectra of (a) 15 nM, (b) 30 nM, (c) 60 nM, (d) 80 nM, (e) 100 nM, and (f) 130 nM. **(B)** Photocurrent responses of (a) 15 nM, (b) 30 nM, (c) 60 nM, (d) 80 nM, (e) 100 nM, and (f) 130 nM. **(C)** PEC linear relationship of HPV 16 DNA detection.

For the detection of HPV 16 DNA, a series of target DNA samples of different concentrations were incubated in the PEC biosensor, and PEC detection was performed accordingly. EIS spectra after this target DNA incubation were also investigated, as depicted in Figure 4 A, and the observed R_et_ value increased gradually with increasing HPV 16 DNA concentrations (15–130 nm), which was predictable because of the increasing steric hindrance. Conversely, the measured PEC response of the biosensor gradually decreased (Figure 4B), exhibiting a PEC quenching phenomenon caused by the addition of T7 exonuclease in the DNA walker cycle process (Scheme 1B) to release the corresponding associated SiW_12_@CdS NPs from the PEC biosensor. Finally, the detection of HPV 16 DNA achieved the expected performance of linear quantitative determination, as shown in Figure 4C. The PEC response had a linear relationship with the concentration of the target DNA, ranging from 15 to 130 nM, with an LOD of 0.8 nM, according to Eq. 1:

$$C_{L}=\frac{K∙S_{b}}{m};$$ (1)

$C_{L}$ : LOD; $S_{b}$ : Blank standard deviation; m : The slope of the calibration curve in the low concentration range that was analyzed; K : Confidence coefficient; the value is 3.

All required photocurrent values are listed in Supplementary Table S1.

### 3.5 Specificity, repeatability, and stability of as-prepared biosensor

**FIGURE 5:**
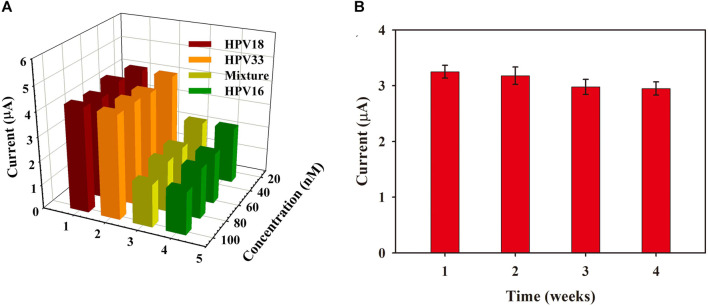
**(A)** Specificity of PEC biosensor against HPV 18 DNA, HPV 33 DNA, HPV16 DNA, and a mixture of all solutions mentioned above (30, 60, 80, and 100 nmol). **(B)** The photocurrent response of the biosensor stored at different times (error bars show S.D., *n* = 3).

Specificity is critical to verify the accuracy and sensitivity of PEC biosensors, indicating their anti-jamming capability. Different HPV subtypes of HPV 18 DNA and HPV 33 DNA were selected as potential disruptors for specific studies of the as-prepared PEC biosensor. As shown in Figure 5 A, the presence of the target HPV 16 DNA (Figure 5A, green column) or target DNA-containing mixture (Figure 5A, yellow-green column) showed an obvious decrease in the PEC response, whereas the highly homologous interference of HPV 18 DNA and HPV 33 DNA would not promote the biological DNA walker cycle process to decrease the photocurrent, indicating the remarkable specificity of the proposed PEC biosensor. For the stability test of the as-prepared PEC biosensor, the fabricated ITO electrodes were preserved at 4°C for 1–4 weeks, and three parallel experiments were conducted every week. As shown in Figure 5B, the PEC response was 90.77% of the initial value after 4 weeks of storage, indicating the significant stability of the as-prepared PEC biosensor. Moreover, within one PEC measurement process, 7 times consecutive light “on/off” cycles were performed to evaluate its repeatability. As shown in Supplementary Figure S2, the photocurrent response exhibited a steady signal with an incredibly small variation, and the relative standard deviation was 6.37%, indicating the distinguished reproducibility of the as-prepared PEC biosensor.

## 4 Conclusion

In summary, a highly sensitive PEC biosensor for detecting HPV 16 DNA, fabricated using SiW_12_@CdS QDs and Au NP/CS gel, was successfully prepared for the first time. The as-prepared SiW_12_@CdS QDs showed an enhanced photoelectric response and high stability after being combined with NP DNA; with the Au NP/CS gel as the first layer on the ITO slides, a series of biochemical DNA primers were incubated to fabricate a multi-site tripodal DNA walker sensing platform coupled with T7 exonuclease. Due to the remarkable conductivity and LSPR of Au NPs, the photosensitivity of the as-prepared biosensor was further improved under $I_{3}^{-}/I^{-}$ electrolytes and avoided the use of other regents toxic to living organisms, and a photocurrent quenching mechanism within the detection process of the as-prepared PEC biosensor was perfectly executed. Finally, under optimized conditions, the as-prepared biosensor protocol demonstrated wide linear ranges (15–130 nM), with an LOD of 0.8 nM and high selectivity, stability, and reproducibility. Furthermore, the proposed PEC biosensor platform offers a reliable and promising pathway for detecting other biological molecules.

## Data Availability

The datasets presented in this study can be found in online repositories. The names of the repository/repositories and accession number(s) can be found in the article/Supplementary Material.
