# Supplementary material for: Photoelectrochemical biosensor based on SiW12@CdS quantum dots for the highly sensitive detection of HPV 16 DNA
Source: Front Bioeng Biotechnol. 2023 Jun 14;11:1193052. doi: 10.3389/fbioe.2023.1193052 (PMC10303914; doi:10.3389/fbioe.2023.1193052)
Supplement: Supplementary file 1 [file Table1.docx]

**Supplementary Material**

**Photoelectrochemical biosensor based on SiW_12_@CdS quantum dots for highly sensitive detection of HPV 16 DNA**

Yao Cheng^1^, Chaoyue Sun^1,2^, Yuhua Chang^3^, Jiayin Wu^1^, Zhihao Zhang^1^, Yunqing Liu^2^, Shenguang Ge^2^, Zhao Li^4^, Xiao Li^5,6^*, Liang Sun^1^* and Dejin ZANG^1^*

1. National Key Laboratory of Advanced Drug Delivery and Release System, NHC Key Laboratory of Biotechnology Drugs (Shandong Academy of Medical Sciences), Key Lab for Rare and Uncommon Diseases of Shandong Province, School of Pharmacy and Pharmaceutical Sciences, Institute of Materia Medica, Shandong First Medical University and Shandong Academy of Medical Sciences, Jinan, China
2. Institute for Advanced Interdisciplinary Research (iAIR), School of Chemistry and Chemical Engineering, University of Jinan, Jinan, China
3. Shandong Provincial Maternal and Child Healthcare Hospital, Jinan, China
4. Suzhou KunTao Intelligent Manufacturing Technology Co., Ltd., Suzhou, China
5. NMPA Key Laboratory for Quality Evaluation of Medical Materials and Biological Protective Devices, Jinan, China
6. Shandong Institute of Medical Device and Pharmaceutical Packaging Inspection, Jinan, China


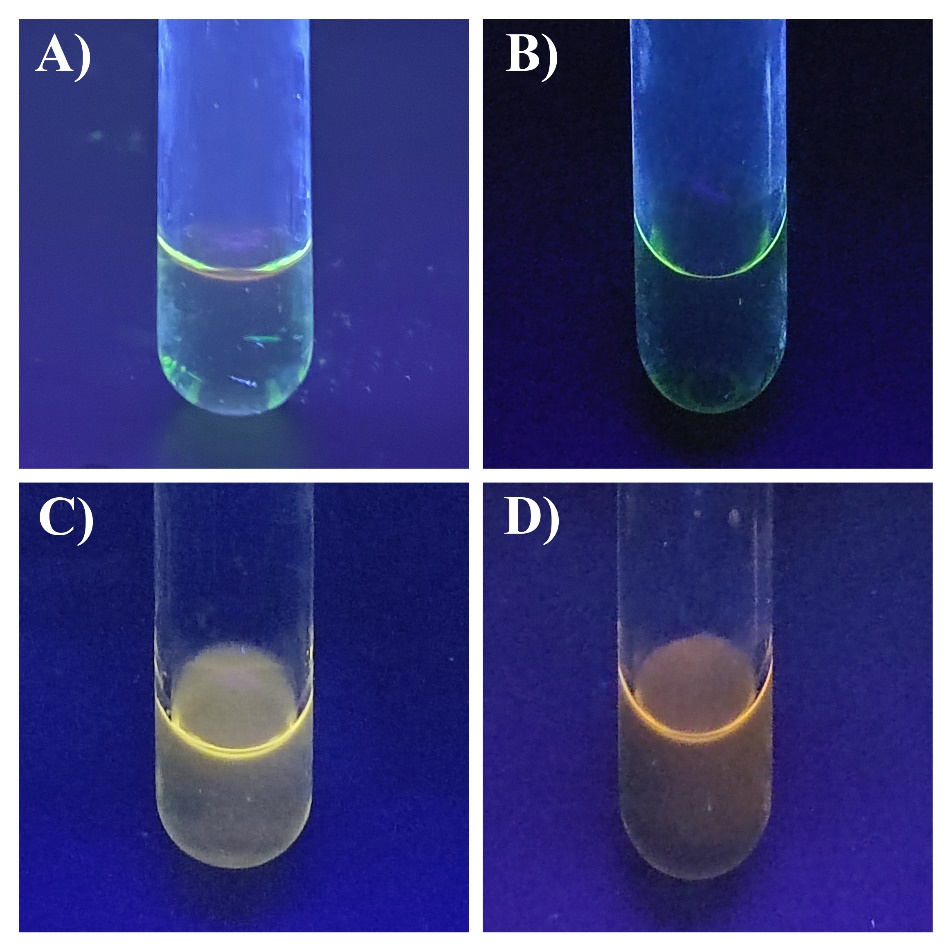


**Figure S1.** 254nm UV-Vis images of A) CdS QDs and B) SiW_12_@CdS QDs.; 365nm UV-Vis images of C) CdS QDs and D) SiW_12_@CdS QDs.

**Table S1.** Photocurrent responses of the PEC biosensor with SiW_12_@CdS QDs (μA) as outmost layer.

| 4.70 | 4.70 | 4.70 | 4.71 | 4.70 |
| --- | --- | --- | --- | --- |
| 4.70 | 4.71 | 4.70 | 4.70 | 4.70 |

*PEC data applied in the calculation of LOD via equation (1) in the maintext.

**
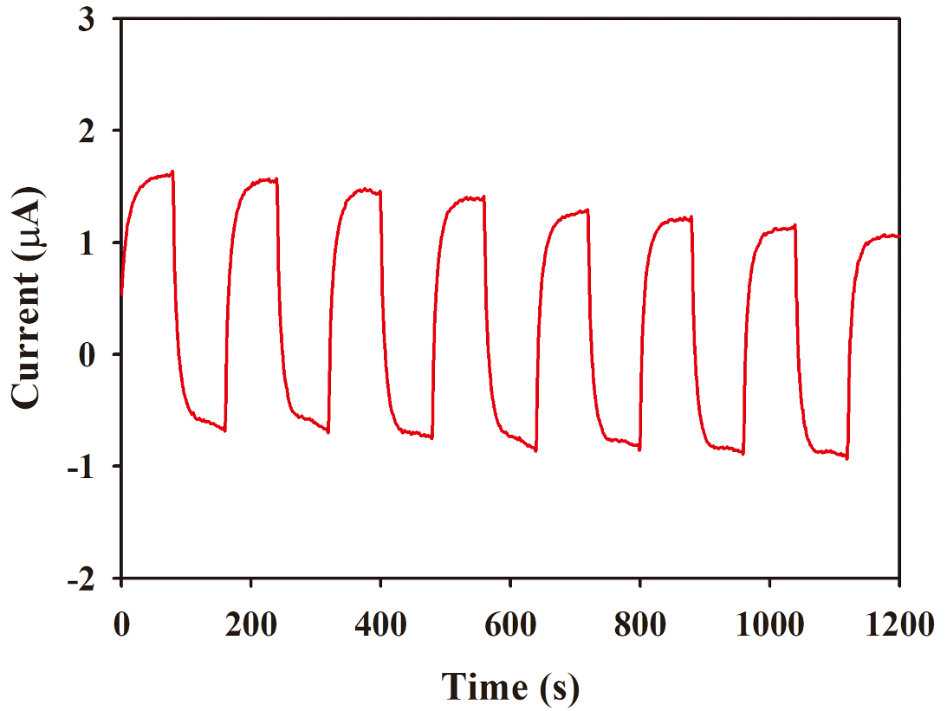
**

**Figure S2.** Successive 7 times of consecutive light “on/off” cycles PEC measurement process of 80 nM HPV16.

**Table S2.** Analytical performances of various methods towards HPV16.

| Analytical method | LOD | Liner range | Years | Refs. |
| --- | --- | --- | --- | --- |
| Electrochemiluminescence | 0.6 nM | 1 nM – 100 nM | 2022 | [1] |
| Electrochemical | 1.3 nM | 8.5 nM - 10.7 μM | 2020 | [2] |
| Photoelectrochemical | 1.6 pM | 0.005 nM - 100 nM | 2021 | [3] |
| Electrochemiluminescence | 0.32 pM | 1 pM - 100 nM | 2021 | [4] |
| Photoelectrochemical | 1.0 pM | 3.0 pM - 600 nM | 2022 | [5] |
| Photoelectrochemical | 0.8 nM | 15 nM -130 nM | 2023 | This work |

**References:**

1. Wang, L.L.; Nie, Y.X.; Zhang X.; Liang, Z.H.; Wang, P.L.; Ma, Q.; Yu, D.H. A novel Eu^3+^ doped polydopamine nano particles/reductive copper particle hydrogel-based ECL sensor for HPV 16 DNA detection. *Microchem J.* **2022**, *181*, 107818. doi: 10.1016/j.microc.2022.107818.
2. Farzin, L.; Sadjadi, S.; Shamsipur M.; Sheibani, S. Electrochemical genosensor based on carbon nanotube/amine-ionic liquid functionalized reduced graphene oxide nanoplatform for detection of human papillomavirus (HPV16)-related head and neck cancer. *J Pharmaceut Biomed.* **2020**, *179*, 112989. doi: 10.1016/j.jpba.2019.112989.
3. Gong, H.X.; Wu, Y.L.; Zeng R.J.; Zeng, Y.Y.; Liu, X.L.; Tang, D.P. CRISPR/Cas12a-mediated liposome-amplified strategy for the photoelectrochemical detection of nucleic acid. *Chem Commun.* **2021**, *57*, 8977-8980. doi: 10.1039/d1cc03743a.
4. Zhao, K.R.; Wang, L.; Liu P.F.; Hang, X.M.; Wang, H.Y.; Ye, S.Y.; Liu, Z.J; Liang, G.X. A signal-switchable electrochemiluminescence biosensor based on the integration of spherical nucleic acid and CRISPR/Cas12a for multiplex detection of HIV/HPV DNAs. *Sensor Actuat B-Chem.* **2021**, *346*, 130485. doi: 10.1016/j.snb.2021.130485.
5. Li, Y.X.; Zeng, R.J.; Wang, W.J.; Xu, J.H.; Gong, H.X.; Li, L.; Li, M.J; Tang, D.T. Size-Controlled Engineering Photoelectrochemical Biosensor for Human Papillomavirus-16 Based on CRISPR-Cas12a-Induced Disassembly of Z-Scheme Heterojunctions. *ACS Sensors.* **2022**, *7*, 1593-1601. doi: 10.1021/acssensors.2c00691.
